# Deep learning identifies synergistic drug combinations for treating COVID-19

**DOI:** 10.1073/pnas.2105070118

**Published:** 2021-09-15

**Authors:** Wengong Jin, Jonathan M. Stokes, Richard T. Eastman, Zina Itkin, Alexey V. Zakharov, James J. Collins, Tommi S. Jaakkola, Regina Barzilay

**Affiliations:** ^a^Computer Science and Artificial Intelligence Laboratory, Massachusetts Institute of Technology, Cambridge, MA 02139;; ^b^Department of Biological Engineering, Synthetic Biology Center, Institute for Medical Engineering and Science, Massachusetts Institute of Technology, Cambridge, MA 02139;; ^c^Broad Institute of MIT and Harvard, Cambridge, MA 02142;; ^d^Division of Preclinical Innovation, National Center for Advancing Translational Sciences, Rockville, MD 20850;; ^e^Abdul Latif Jameel Clinic for Machine Learning in Health, Massachusetts Institute of Technology, Cambridge, MA 02139;; ^f^Wyss Institute for Biologically Inspired Engineering, Harvard University, Boston, MA 02115;; ^g^Harvard-MIT Program in Health Sciences and Technology, Cambridge, MA 02139

**Keywords:** deep learning, drug discovery, drug synergy, SARS-CoV-2

## Abstract

Effective treatments for COVID-19 are urgently needed. However, discovering single-agent therapies with activity against severe acute respiratory syndrome coronavirus 2 (SARS-CoV-2) has been challenging. Combination therapies play an important role in antiviral therapies, due to their improved efficacy and reduced toxicity. Recent approaches have applied deep learning to identify synergistic drug combinations for diseases with vast preexisting datasets, but these are not applicable to new diseases with limited combination data, such as COVID-19. Given that drug synergy often occurs through inhibition of discrete biological targets, here we propose a neural network architecture that jointly learns drug−target interaction and drug−drug synergy. The model consists of two parts: a drug−target interaction module and a target−disease association module. This design enables the model to utilize drug−target interaction data and single-agent antiviral activity data, in addition to available drug−drug combination datasets, which may be small in nature. By incorporating additional biological information, our model performs significantly better in synergy prediction accuracy than previous methods with limited drug combination training data. We empirically validated our model predictions and discovered two drug combinations, remdesivir and reserpine as well as remdesivir and IQ-1S, which display strong antiviral SARS-CoV-2 synergy in vitro. Our approach, which was applied here to address the urgent threat of COVID-19, can be readily extended to other diseases for which a dearth of chemical−chemical combination data exists.

## Significance

COVID-19 has caused more than 2.5 million deaths worldwide. It is imperative that we develop therapies that can mitigate the effect of the disease. While searching for individual drugs for this purpose has been met with difficulties, synergistic drug combinations offer a promising alternative. However, the lack of high-quality training data pertaining to drug combinations makes it challenging to use existing machine learning methods for effective novel combination prediction tasks. Our proposed approach addresses this challenge by leveraging additional readily available data, such as drug−target interactions, thus enabling an effective in silico search for synergistic combinations against SARS-CoV-2.

Combination therapies have been shown to be more effective than single drugs for multiple diseases such as HIV (1) and infections caused by *Mycobacterium tuberculosis* (2). Synergistic combinations can improve both therapeutic potency and efficacy, either achieving stronger therapeutic effects and/or decreasing the required dose, thereby reducing side effects. To address the COVID-19 pandemic, and future pandemics, finding useful combinations of approved molecules has an additional benefit over discovering and developing an entirely novel single-agent therapy: reduced time to clinical adoption. Approved drugs are readily available at scale, have well-studied toxicity profiles, and may be used off-label in extenuating circumstances. Collectively, these considerations highlight the benefits of discovering new synergistic drug combinations for treating COVID-19.

Exploring the space of combinations via high-throughput screening of even midsized chemical libraries is prohibitive due to the exceedingly large number of unique chemical combinations. Therefore, in silico screening based on various computational methods is an appealing alternative (3, 4). For example, Bobrowski et al. (5) used knowledge-based methods to generate candidate drug combinations and experimentally validated their antiviral severe acute respiratory syndrome coronavirus 2 (SARS-CoV-2) synergies. Cheng et al. (6) developed a biological network proximity measure to predict drug synergy for hypertension and cancer. Prior work has applied various machine learning techniques for synergy prediction (7–9), including deep learning approaches (10–12). Indeed, Preuer et al. (10) trained a deep neural network on a large oncology screen (13) and demonstrated the advantage of deep learning over standard machine learning models such as RFs and SVMs.

Unfortunately, there are two primary challenges that prevent one from applying existing deep learning approaches to predict therapeutic chemical combinations for emerging pathogens such as SARS-CoV-2. First, deep neural networks require a large amount of training data with measured synergy scores. While such data are readily available for some diseases such as cancer (13) (more than 20,000 combinations), the amount of SARS-CoV-2 drug combination data (5) is very limited (less than 200 combinations). Second, even the largest combination screen for cancer (14) covers only around 100 different molecules, since the number of pairwise combinations grows quadratically. This significantly limits a model’s ability to generalize to new chemical spaces outside of the training set. Therefore, we posit that a model should incorporate additional information besides molecular structures in order to accurately predict new synergistic drug combinations.

The main contribution of this paper is a deep learning architecture, which we call ComboNet, that jointly models molecular structure, as well as biological targets, for the purpose of predicting synergistic drug combinations. Our hypothesis is that, by explicitly modeling interactions between drugs and biological targets, we can significantly decrease the dependence on combination synergy data. Indeed, uniquely, relative to previous approaches (3, 4, 9, 15, 16) using drug−target interaction (DTI) as fixed descriptors, ComboNet learns to predict DTI from molecular structures, which is advantageous since a large proportion of compounds in our training dataset have incomplete DTI information.

The ComboNet architecture consists of two components. The first component is a graph convolutional network (GCN) (17) that learns a continuous representation of a molecule. This representation contains both structural features of the molecule and predicted targets (i.e., what biological targets may interact with the molecule). Specifically, the biological targets in our training dataset include SARS-CoV-2 3CL protease, angiotensin-converting enzyme 2 (ACE2), and 31 host targets that physically interact with viral proteins (18). The GCN learns to predict the most likely targets, using data collected from the Chemical Database of European Molecular Biology Laboratory (ChEMBL) (19) and US National Center for Advancing Translational Sciences (NCATS) OpenData portal (20). The 31 host targets included in ComboNet are only a subset of the 332 targets that physically interact with SARS-CoV-2 (18). Other targets were excluded because they lack available DTI data.

The second component of ComboNet models target−disease association. It is a linear function that learns how biological targets and structural features of molecules are related to antiviral activity and synergy. It is trained on NCATS single-agent SARS-CoV-2 cytopathic effect (CPE) assay data (21) and available drug combination assays (22). In short, ComboNet predicts drug combination synergy by modeling structural features of both compounds and biological targets.

Herein, we evaluated ComboNet on a hold-out test set (5) of 71 drug combinations with measured anti−SARS-CoV-2 synergy in vitro. Our model achieves 0.82 receiver operating characteristic−area under the curve (ROC-AUC) using ∼200 drug combination data for training, with specificity = 0.75 and sensitivity = 0.80. We additionally applied ComboNet to in silico repurposing of existing drugs and experimentally tested 30 drug combinations. From this empirical set of 30 tested combinations, we discovered two drug combinations (remdesivir and reserpine; remdesivir and IQ-1S) with strong synergy in vitro. In general, ComboNet represents an advance toward predicting novel chemical−chemical synergy for instances where minimal combination training data exist.

## Results

**Fig. 1.**
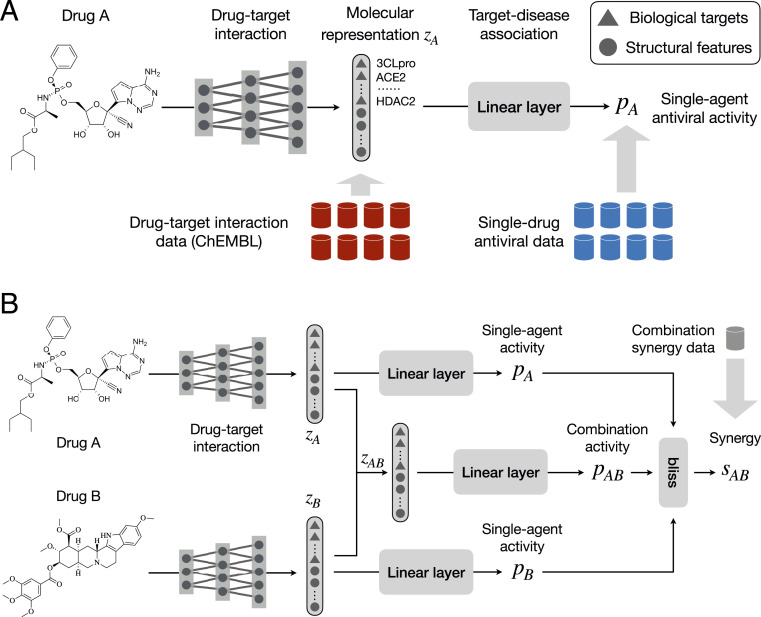
ComboNet for synergistic drug combination discovery. (*A*) ComboNet is composed of two networks: a DTI and a target−disease association network. The antiviral effect of a single drug $p_{A}$ is predicted from its representation $z_{A}$. The vector $z_{A}$ characterizes the DTI features of drug A. (*B*) The antiviral effect of a combination is predicted from its representation $z_{AB}$, which is computed from the molecular representations of each individual drug $z_{A},z_{B}$. ComboNet is trained on drug combination synergy, single-drug antiviral activity, and DTI data.

Fig. 1 provides an overview of the network architecture. It is composed of a DTI network and target−disease association network. These are trained to accomplish three tasks: 1) predict the interaction between a drug and a set of K biological targets $\left\{t_{1},\ldots ,t_{K}\right\}$ related to the disease of interest, 2) predict a drug’s intrinsic antiviral activity, and 3) predict the synergy of two drugs. The latter two tasks depend on both the predicted biological targets and structural features of input molecules.

### Drug−Target Interaction Prediction.

The DTI network is trained to predict whether a drug binds to a biological target. The DTI training data are compiled from ChEMBL, including K biological targets related to the indication or pathogen of interest—in our case, SARS-CoV-2. Each DTI dataset consists of a list of molecules and their binary DTI labels (positive/negative). A positive label means the binding affinity (e.g., half-maximal effective concentration [${EC}_{50}$]) of a molecule to a target is below a certain threshold. In terms of SARS-CoV-2 biological targets, we consider both viral proteases and host proteins involved in viral infection. The replication of SARS-CoV-2 requires the processing of a chymotrypsin-like protease (3CLpro). It is known that SARS-CoV-2 entry into host cells depends on ACE2 and TMPRSS2 (23). Furthermore, Gordon et al. (18) identified 332 human proteins that physically interact with SARS-CoV-2.

The DTI training data for these targets are collected from various sources. NCATS conducted a high-throughput screen of 10,442 compounds with measured 3CLpro enzymatic activity (24). NCATS also released two high-throughput screens of 3,285 molecules with measured ACE2 enzymatic activity (25) and inhibition against Spike−ACE2 protein−protein interaction (26). Among the 332 human proteins, we selected 31 targets based on their DTI data availability in ChEMBL (19). Other targets were excluded due to a lack of existing DTI data.

We parameterize the DTI network as a directional message passing neural network (DMPNN) (17). Each compound is characterized as a graph, whose nodes and edges correspond to its atoms and bonds. The DMPNN applies a series of message passing steps to aggregate information from neighboring atoms and bonds to build a continuous vector representation $z_{A}$ of drug A. We divide $z_{A}$ into two vectors $z_{A}=z_{A}^{covid}⊕z_{A}^{struct}$ (⊕ represents vector concatenation). The $z_{A}^{covid}$ represents the predicted interaction between drug A and biological targets related to SARS-CoV-2. Each element $z_{A,i}^{covid}\in \left[0,1\right]$ indicates the probability of drug A interacting with a target $t_{i}$. The $z_{A}^{struct}$ represents the structural features of drug A learned automatically from its molecular structure. Each element $z_{A,i}^{struct}\in \left[0,1\right]$ is output from a sigmoid activation function.

**Fig. 2.**
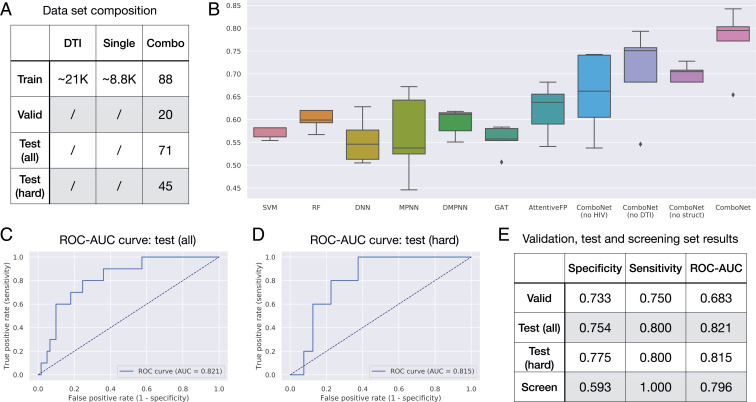
In silico evaluation of ComboNet (*A*) The training, validation, and test set composition for SARS-CoV-2. (*B*) Results on SARS-CoV-2 drug combination test set. Our full ComboNet model outperforms all other baselines. (*C*) ROC-AUC plot of ComboNet ensemble on the entire test set. (*D*) ROC-AUC plot of ComboNet ensemble on the hard drug combinations with at least one new drug. (*E*) Statistical characteristics of ComboNet ensemble for all the datasets, where “screen” refers to the top 30 candidates we experimentally tested.

We propose to include these structural features to increase the modeling power when target information is incomplete. Among the 332 human proteins, only 31 of them have associated DTI data, and the other 300 targets cannot be included in the model. Moreover, our biological understanding of emerging pathogens is continuously involving. Including these structural features allows the model to complement any missing biological information needed for antiviral activity and synergy prediction. Indeed, we observe a decrease in synergy prediction accuracy when these structural features are removed (Fig. 2).

### Single-Agent Activity Prediction.

We train the entire ComboNet to predict the antiviral activity of single drugs. The single-agent training set is a collection of molecules with their antiviral activity labels (positive/negative). A positive label indicates that a drug inhibits the viral replication. The DTI network is trained to extract useful features from molecular structures for antiviral activity prediction. The target−disease association network f learns how to associate the biological targets and learned structural features of molecules to antiviral activity. It is parameterized as a simple linear layer with sigmoid activation function σ(⋅). The antiviral activity of a single drug A is predicted as

$$p_{A}=f\left(z_{A}\right)=\sigma \left(w^{⊤}z_{A}+b\right).$$ [1]

The model is trained on SARS-CoV-2 single-agent antiviral activity data using a CPE assay (21) in VeroE6 cells. It contains ∼8,800 compounds with 320 hits (${EC}_{50}\leq 10\;\mu$M).

### Synergy Prediction.

In addition, we train the entire ComboNet to predict drug−drug synergy. The training set for this task is a list of pairwise drug combinations and their synergy labels (synergistic/nonsynergistic). Different from the previous two tasks, inputs to the model become two molecules instead of one. Given a pairwise drug combination (A,B), the DTI network outputs a continuous vector representation $z_{AB}$ by combining their individual representations $z_{A},z_{B}$. The combined vector characterizes how the two drugs interact via their individual biological targets. It is then fed into the target−disease association network to predict its synergy based on Bliss scores (27).

We adopt the Bliss score (27) to predict synergy of a drug combination (Fig. 1*B*). Suppose the individual antiviral effect of drugs A and B are $p_{A},p_{B}$. The expected activity of a combination (A,B) is defined as $e_{AB}=p_{A}+p_{B}-p_{A}p_{B}$. A drug combination is determined to be synergistic if its actual activity $p_{AB}>e_{AB}$. Thus, we define its synergy score as

$$s_{AB}=p_{AB}-e_{AB}=p_{AB}-p_{A}-p_{B}+p_{A}p_{B},$$ [2]

where the antiviral activity $p_{AB}$ of a drug combination (A,B) is predicted as

$$p_{AB}=f\left(z_{AB}\right)=\sigma \left(w^{⊤}z_{AB}+b\right).$$ [3]

The remaining question is how to compute the molecular representation $z_{AB}$ for a drug combination. Since we model drug synergy using Bliss scores, we introduce a Bliss layer to compute the representation of a drug combination. Let $z_{A},z_{B}$ be the learned features of drugs A and B. The representation $z_{AB}$ of a combination (A,B) is defined as

$$z_{AB}=z_{A}+z_{B}-z_{A}⊙z_{B},$$ [4]

where ⊙ stands for element-wise multiplication. With this aggregation scheme, a drug combination benefits the most when two drugs interact with different targets. For instance, suppose only drug A interacts with target $t_{i}$ (e.g., $z_{A,i}=0.9,z_{B,i}=0$); the combination still interacts with target $t_{i}$ as $z_{AB,i}=0.9$.

The SARS-CoV-2 drug combination training data came from three data sources. NCATS performed two combination assays (5, 22) in VeroE6 cells, which contained 160 two-drug combinations in total. Riva et al. (28) also analyzed synergy between remdesivir and 20 hit molecules identified from their high-throughput screen in VeroE6 cells.

### Multidisease Training.

The drug combination data of emerging pathogens are inherently limited. To address the low-resource challenge, it is helpful to utilize data from multiple diseases as a source of supervision. For example, we can utilize existing HIV drug combination data to improve the model performance. Indeed, prior work (18) has shown significant interactome similarity between HIV and SARS-CoV-2. With multidisease training, the molecular representation $z_{A}$ contains three parts $z_{A}^{covid},z_{A}^{hiv},z_{A}^{struct}$. Features in $z_{A}^{hiv}$ correspond to the interaction between drug A and HIV-relevant biological targets. Since each disease operates on different targets, we create two target−disease association networks, $f^{covid}$ and $f^{hiv}$. The SARS-CoV-2 and HIV antiviral activity is computed as $f^{covid}\left(z_{A}^{covid}⊕z_{A}^{struct}\right)$ and $f^{hiv}\left(z_{A}^{hiv}⊕z_{A}^{struct}\right)$, respectively (⊕ represents vector concatenation).

In terms of HIV targets, we consider three viral proteases (HIV-1 protease, integrase, and reverse transcriptase) and three host proteins involved in viral entry (CCR5, CXCR4, and CD4) (29). We compiled DTI data for these six targets from ChEMBL. The HIV single-agent activity data came from a National Cancer Institute (NCI) anti-HIV assay (30). It includes ∼35,000 compounds with 309 active hits (${EC}_{50}\leq 1\;\mu$M). The HIV combination data (1) contain 114 drug combinations with measured synergy outcomes against HIV.

### Training Objective.

The ComboNet is trained to minimize a weighted average of three losses $L=\lambda_{DTI}\ell_{DTI}+\lambda_{S}\ell_{S}+\ell_{C}$, where $\lambda_{DTI},\lambda_{S}$ are hyperparameters, and $\ell_{DTI},\ell_{S},\ell_{C}$ are the training losses on the DTI, single-agent, and drug combination data. The weighted loss allows us to optimize the entire model with a single forward−backward pass in each gradient update.

### Model Evaluation.

We evaluate our model’s performance at predicting SARS-CoV-2 chemical synergy. Our training, validation, and test sets are summarized in Fig. 2*A*. Specifically, our validation set contains 20 drug combinations from Riva et al. (28), and our test set contains 71 drug combinations from Bobrowski et al. (5). The training set contains 88 SARS-CoV-2 drug combinations from NCATS (22) as well as the DTI and single-agent antiviral activity data for SARS-CoV-2 and HIV. We note that 63.4% (45/71) of the drug combinations in the test set involved at least one new drug that did not appear in the training set.

### Baselines.

To test the effectiveness of ComboNet, we compare our approach with seven baselines: a random forest (RF), support vector machine (SVM), feed-forward neural network (DNN), and four state-of-the-art graph neural network architectures, including MPNN (31), DMPNN (17), graph attention network (GAT) (32), and AttentiveFP (33). All baselines are trained on SARS-CoV-2 combination data only, while ComboNet is trained on additional HIV, DTI, and single-agent data.

The input to RF and SVM is the sum of ECFP4 fingerprints of the two drugs so that the model is permutation invariant; that is, it outputs the same value for drug pairs (A,B) and (B,A). The DNN and graph neural network baselines predict the synergy of drugs A and B as $p_{AB}=g\left(\phi \left(A\right)+\phi \left(B\right)\right)$, where g is a feed-forward network with one hidden layer. For DNN, the input to ϕ is the ECFP4 fingerprint of drugs A and B. For MPNN, DMPNN, GAT, and AttentiveFP, the input to ϕ is the molecular graph of A and B. We sum the two vectors ϕ(A)+ϕ(B) instead of concatenating them so that the model is permutation invariant. We also use the same ϕ to encode drugs A and B to ensure permutation invariance.

Moreover, we evaluate the following ComboNet variants to study the importance of different training data: 1) ComboNet (no HIV), a model trained without HIV data; 2) ComboNet (no DTI), a model trained on all training data except the DTI data; 3) ComboNet (no struct), a model trained on all training data but the structural features are disabled; and 4) ComboNet, a model trained on all the training data.

Additional ablation studies and interpretability analysis are provided as *SI Appendix*.

### Synergy Prediction Accuracy.

The results of synergy prediction are shown in Fig. 2*B*. We compute the ROC-AUC of each method averaged across five independent runs. The test ROC-AUC of ComboNet is 0.773±0.064, which is significantly higher than the RF, SVM, DNN, and DMPNN baselines. Among all baseline methods, AttentiveFP achieves the best ROC-AUC of 0.621±0.050. The Wilcoxon *P* value between ComboNet and AttentiveFP is 0.028.

We then took five independently trained ComboNet models as an ensemble model. Ensembling is a standard machine learning technique to improve model performance, where we train five copies of ComboNet with different random initialization and average their predictions. The ensemble model achieves 0.821 ROC-AUC on the test set (Fig. 2*C*), which is higher than a single ComboNet model.

We further adopt a “compounds out” strategy (34) to evaluate the model in terms of novel combination prediction. Specifically, we select 45 combinations from the test set that involve at least one new drug that has not appeared in the training set. The average Tanimoto similarity between these 45 combinations and the training set is low: ∼0.22. Thus, these instances are significantly harder to predict, and require the model to extrapolate beyond drugs in the training set. Remarkably, the ensemble model achieves similar performance on these difficult instances, with ROC-AUC = 0.815 (Fig. 2*D*). This result shows that ComboNet generalizes well to novel drug combinations.

### Benefit of DTI and HIV Data.

We further conduct ablation studies to understand the importance of different model design choices (Fig. 2*B*). We find the test ROC-AUC decreases to 0.658±0.079 if the HIV data are removed (ComboNet, no HIV). Likewise, the test ROC-AUC drops to 0.706±0.088 when we remove the DTI data (ComboNet, no DTI). This confirms the advantage of training with DTI data and additional viral diseases.

### Benefit of Structural Features.

The test ROC-AUC decreases to 0.701±0.017 if we remove the structural features (ComboNet, no struct) (Fig. 2*B*). This highlights the advantage of using structural features to complement missing biological targets.

### Screening Predicted Drug Combinations.

We applied the ComboNet ensemble to predict the synergy of novel drug combinations in the NCATS compound library. We considered pairwise combinations between 153 relatively potent drugs with half-maximal inhibitory concentration (${IC}_{50}$) less than 30 μM. This resulted in ∼11,600 combinations, which were ranked according to predicted synergy scores. We selected the top 30 candidates and experimentally tested them in a SARS-CoV-2 CPE assay, which measures the ability of compounds to reverse the viral-induced CPE in Vero E6 host cells. In this assay, viral infection and replication lead to a loss of host cell viability. Compounds with antiviral activity protect cells from the virus, thereby maintaining viability.

**Fig. 3.**
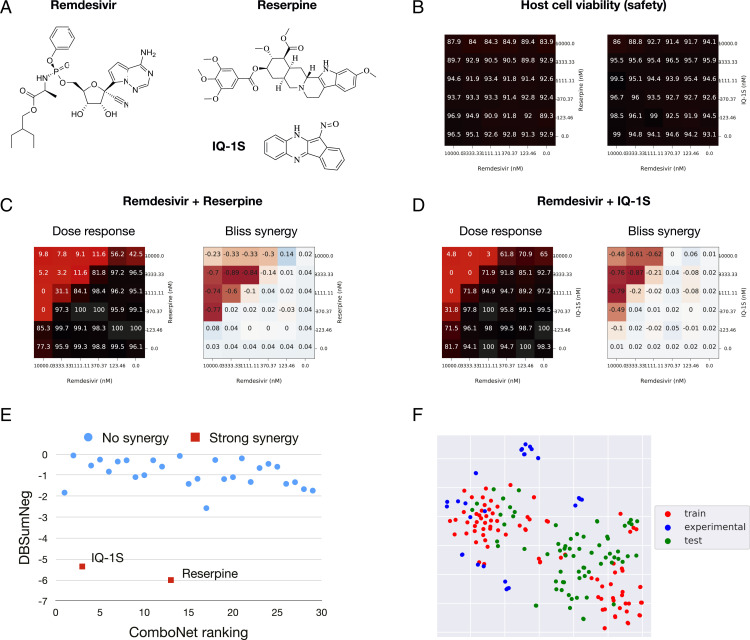
Discovery of synergistic drug combinations for SARS-CoV-2 (*A*) Two drug combinations are discovered by our model: remdesivir + reserpine and remdesivir + IQ-1S. (*B*) Host cell viability matrices show the two drug combinations have low cytotoxicity. (*C*) Dose–response and Bliss synergy matrices of remdesivir + reserpine. Numbers in the dose–response matrix stand for viral infection rate. Numbers in the Bliss synergy matrix stand for synergy score. Both are the lower the better. (*D*) Dose–response and Bliss synergy matrices of remdesivir + IQ-1S. (*E*) The correlation between predicted ranking and DBSumNeg score (lower DBSumNeg means more synergistic). (*F*) The t-distributed stochastic neighbor embedding visualization (36) of the chemical space explored across the training set, test set, and experimentally validated combinations.

The synergy of these combinations was assessed based on the Delta Bliss Sum Negative (DBSumNeg) score (35). Excitingly, from this set of 30 empirically tested predicted combinations, we identified two drug combinations—remdesivir and reserpine, as well as remdesivir and IQ-1S (Fig. 3*A*)—with strong synergy in vitro (DBSumNeg ≤−5). Importantly, we also verified that these two drug combinations have low cytotoxicity (Fig. 3*B*). Their dose–response and Bliss synergy matrices are reported in Fig. 3 *C* and *D*. The ComboNet ranking of the 30 drug combinations is reported in Fig. 3*E*. As visualized in Fig. 3*F*, the chemical spaces explored across the training/test sets and experimentally validated combinations are quite similar.

Reserpine is Food and Drug Administration−approved drug primarily used as a peripheral antihypertensive. It has a moderate potency against SARS-CoV-2, with ${IC}_{50}$ = 11.2 μM in Vero E6 cells (21) and ${IC}_{50}$ = 6.4 μM in HeLa-ACE2 cells (37). IQ-1S is a JNK inhibitor with *K*_d_ = 87, 360, and 390 nM for JNK3, JNK2, and JNK1, respectively. It demonstrated an ${IC}_{50}$ = 6.3 μM against SARS-CoV-2 in a Vero E6 cell CPE assay.

## Discussion

In this study, we developed ComboNet for chemical synergy prediction against SARS-CoV-2. ComboNet has two components: a DTI network and a target−disease association network. The model architecture is designed to utilize additional DTI data and single-agent antiviral activity data. Although our synergy training set contains only 88 drug combinations, ComboNet achieves 0.82 test ROC-AUC, while standard deep learning methods struggle to reach 0.6 ROC-AUC. We then performed virtual screening on 11,600 candidate drug combinations using ComboNet, empirically tested 30 of these predictions, and identified and validated two drug combinations with strong synergy in vitro.

Recently, deep learning approaches have demonstrated success in drug discovery (38). A common approach is to train a deep neural network to perform virtual screening over chemical libraries in silico and prioritize compounds among the top predictions for laboratory testing. In order to provide accurate rankings, these models require a fair amount of training data (e.g., more than 2,000 compounds) to predict biological activities. Unfortunately, such data are typically not available for an emerging pathogen like SARS-CoV-2. Therefore, it is crucial to leverage additional biological knowledge of these pathogens to complement the limited task-specific data.

ComboNet is motivated by the recent success of GCNs in molecular property prediction (17, 31, 39). Most of these models learn molecular representations based on chemical structures alone and do not explicitly model biological interaction. On the other hand, while traditional cheminformatics tools have modeled DTI for property prediction (6, 40), most of these methods do not leverage chemical structures like the GCNs. ComboNet seeks to incorporate the merit of both approaches in a unified deep learning architecture.

The role of the structural features learned by GCNs is to mitigate the incompleteness of biological information. An interesting future direction is to make these structural features biologically interpretable. For example, we can speculate that a structural feature in molecular representation may correspond to a biological target if they are activated (or inactivated) by the same set of molecules. This may allow us to automatically identify new targets related to a specific disease.

## Materials and Methods

### ComboNet Architecture.

Our ComboNet implementation builds on the Chemprop software (17). The atom features include atomic number, degree, formal charge, chirality, number of bonded hydrogens, hybridization, aromaticity, and atomic mass. The bond features include bond type (single/double/triple/aromatic), conjugation, ring membership, and stereochemistry. The model applies a series of message passing steps to learn atom representations. In each step of message passing, each atom’s featurization is updated by summing the incoming messages concatenating the current atom’s featurization, and then applying a single neural network layer with nonlinear activation. After a fixed number of message passing steps, the learned atom representations are summed to produce a single molecular representation $z_{A}$. We set the dimension of vector representation |z|=100. The Chemprop software is open source and available at https://github.com/chemprop/chemprop.

### Baseline Implementation.

We run the RF and SVM baselines using the “sklearn_train.py” script in Chemprop. The ECFP4 fingerprint is calculated using RDKit (41), with dimensions equal to 2,048. We run the MPNN and DMPNN baseline using the “train.py” script in Chemprop with “–atom_messages” option. We implemented the GAT architecture in the Chemprop software since it is not directly available. The AttentiveFP implementation is copied from https://github.com/OpenDrugAI/AttentiveFP.

### Biological Targets.

The SARS-CoV-2 targets include 3CLpro, PLpro, ACE2 enzymatic assay, Spike−ACE2 protein interaction assay, and 31 host proteins. The UniProt IDs of the SARS-CoV-2 host proteins are O60885, O96028, P00750, P05556, P06280, P09884, P12268, P14735, P17612, P19784, P21964, P25440, P26358, P27448, P33527, P48556, P55085, P55789, P62873, P67870, Q13443, Q13546, Q7KZI7, Q8WTV0, Q92769, Q99720, Q9H773, Q9H7Z7, Q9P0L2, Q9UHD2, and Q9UHI8. The HIV targets are HIV-1 protease (CHEMBL4296312), integrase (CHEMBL2366505), reverse transcriptase (CHEMBL247, CHEMBL2366516), CCR5 (CHEMBL274), CXCR4 (CHEMBL2107), and CD4 (CHEMBL2754).

### Data Curation.

The SARS-CoV-2 DTI data are downloaded from Gordon et al. (18). The original DTI data are turned into a binary classification dataset as follows. We define that a molecule interacts with a target if its binding affinity is below a threshold τ. Following IDG’s activity thresholds, we set τ= 33 nM for kinases, τ= 100 nM for GPCR proteins, and τ=1 μM for other proteins. We set τ=10 μM for NCATS 3CLpro and ACE2 assays. The HIV DTI data are downloaded from ChEMBL and binarized in the same way.

The COVID-19 single-agent data were downloaded from NCATS OpenData Portal. It contained ∼8,800 unique compounds. A compound is active to SARS-CoV-2 if its ${EC}_{50}\leq 10\;\mu$M. The HIV single-agent data were downloaded from NCI (30). It includes ∼35,500 unique compounds. A compound is active to HIV if its ${EC}_{50}\leq 1\;\mu$M.

The SARS-CoV-2 drug combination training data come from NCATS OpenData Portal. A drug combination is synergistic if its DBSumNeg score is less than −4. The validation and test set come from Riva et al. (28) and Bobrowski et al. (5), respectively. The synergy labels were already binarized in the original data. The HIV drug combination data come from Tan et al. (1), whose synergy label was already binarized by Bliss synergy calculation.

All datasets are provided as *SI Appendix*.

### SARS-CoV-2 CPE Assay.

Vero E6 cells were premixed with SARS-CoV-2 virus for 5 min to 10 min, and then dispensed into assay-ready plates (predispensed with compounds and controls). Cells and virus were incubated with compounds for 72 h, and then viability was assayed by Vero E6 host cell ATP content (using Promega CellTiterGlo). Sixty nanoliters per well of each compound in dimethyl sulfoxide (DMSO) was spotted into 384-well assay plates by acoustic dispensing. Five microliters per well of media was dispensed into plates (minimal essential medium, 1% Pen/Strep/GlutaMax, 1% Hepes, 2% heat-inactivated fetal bovine serum).

We then dispensed 25 μ per well of Vero E6 cells inoculated with SARS-CoV-2 (USA-WA1/2020) at a multiplicity of infection of 0.002 suspended in media. Final cell density was 4,000 cells per well. Assay plates were incubated for 72 h at 37 °C, 5% CO_2_, 90% humidity. Plates were incubated for 10 min at room temperature, and luminescence signal was read on a PerkinElmer Envision plate reader. Finally, data were normalized to the negative control (DMSO) and positive controls (cells without virus and Calpain inhibitor IV) for each plate.

## Supplementary Material

Supplementary File

Supplementary File

Supplementary File

Supplementary File

Supplementary File

Supplementary File

Supplementary File

Supplementary File

## Data Availability

All study data are included in the article and *SI Appendix*.

## Change History

October 16, 2021: The Acknowledgments have been updated.
