# Supplementary material for: Deep learning identifies synergistic drug combinations for treating COVID-19
Source: Proc Natl Acad Sci U S A. 2021 Sep 15;118(39):e2105070118. doi: 10.1073/pnas.2105070118 (PMC8488647; doi:10.1073/pnas.2105070118)
Supplement: Supplementary File [file pnas.2105070118.sapp.pdf]

PNAS
www.pnas.org

Supplementary Information for
Deep learning identifies synergistic drug combinations for treating COVID-19

Wengong Jin, Jonathan M. Stokes, Richard T. Eastman, Zina Itkin, Alexey V. Zakharov, James J. Collins, Tommi S. Jaakkola, Regina Barzilay

Corresponding author: Wengong Jin
Email: wengong@csail.mit.edu

This PDF file includes:

Supplementary text
Figures S1
Legends for Datasets S1 to S7
SI References

Other supplementary materials for this manuscript include the following:

Datasets S1 to S7

Supplementary Information Text

Ablation study on validation set. In our experiments, we used a validation set of 20 SARS-CoV-2 drug combinations for simplicity. One potential disadvantage of this approach is that the small validation set may cause bias in model selection. To address this issue, we construct a larger validation set by randomly sampling 20% of the DTI and single-agent data for validation, in addition to the 20 drug combinations. In each training epoch, we measure a model's accuracy on the DTI validation data, single-agent validation data and drug combination validation data. We select a model checkpoint with the highest validation accuracy averaged across the three validation datasets. ComboNet achieves 0.766 ± 0.035 ROC-AUC under the new validation strategy, which is similar to the original result. This shows that the original validation set does not affect model selection.

Interpretability analysis. Besides the ablation study of different model components, it is also important to analyze the rationale behind ComboNet predictions, which explains why a drug combination is predicted as synergistic. Specifically, we analyze the combination of nitazoxanide and remdesivir, which has been reported to have high synergy against SARS-CoV-2 in Bobrowski et al. [1]. We apply a gradient attribution method [2] to analyze why this drug combination is predicted as synergistic. In Figure S1a, we show the gradient of predicted synergy score s_{AB} with respect to learned DTI vectors $\mathbf{z}_A$ and $\mathbf{z}_B$ (A = nitazoxanide, B = remdesivir). A large gradient $\frac{\partial s_{AB}}{\mathbf{z}_{A,i}^{covid}}$ indicates a strong correlation between synergy and drug A's interaction with a biological target $\mathrm{t_i}$. We observe a significant positive correlation between SARS-CoV-2 synergy and MARK2 while ADAMTS1 and NSD2 are negatively correlated with synergy.

The ComboNet architecture also allows us to investigate how each biological target is related to antiviral activity. In the target-disease association network f, each element in the weight vector w (see Equation 3) learns a target-disease association score that describes the correlation between a biological target and antiviral activity. In Figure S1a, we plot the learned target-disease association scores for each biological target related to SARS-CoV-2. We found that MARK2, GLA, IDE, MARK3, and HDAC2 receives the highest score, suggesting their important role in SARS-CoV-2 antiviral activity.

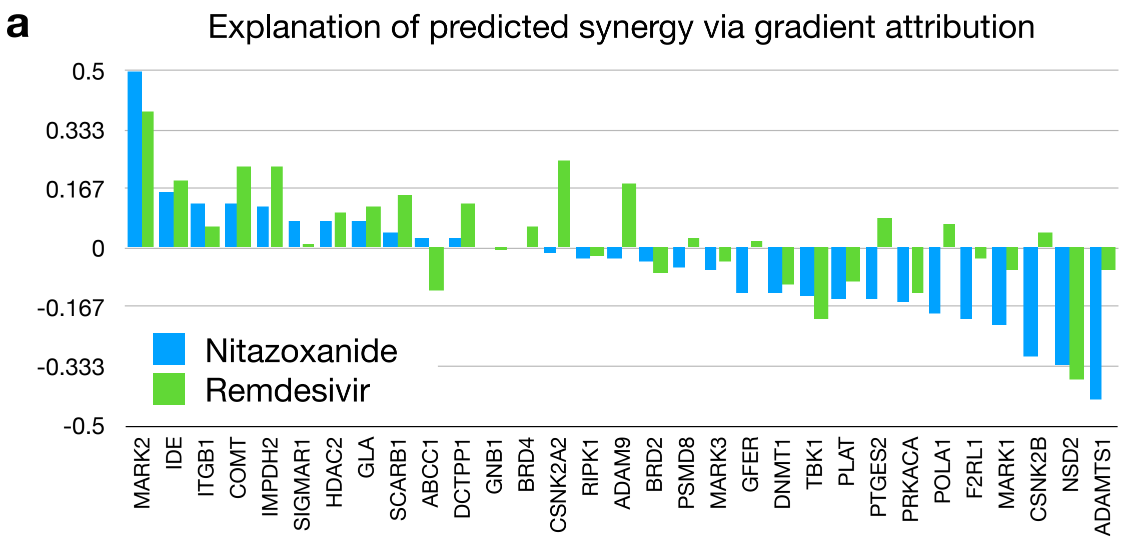

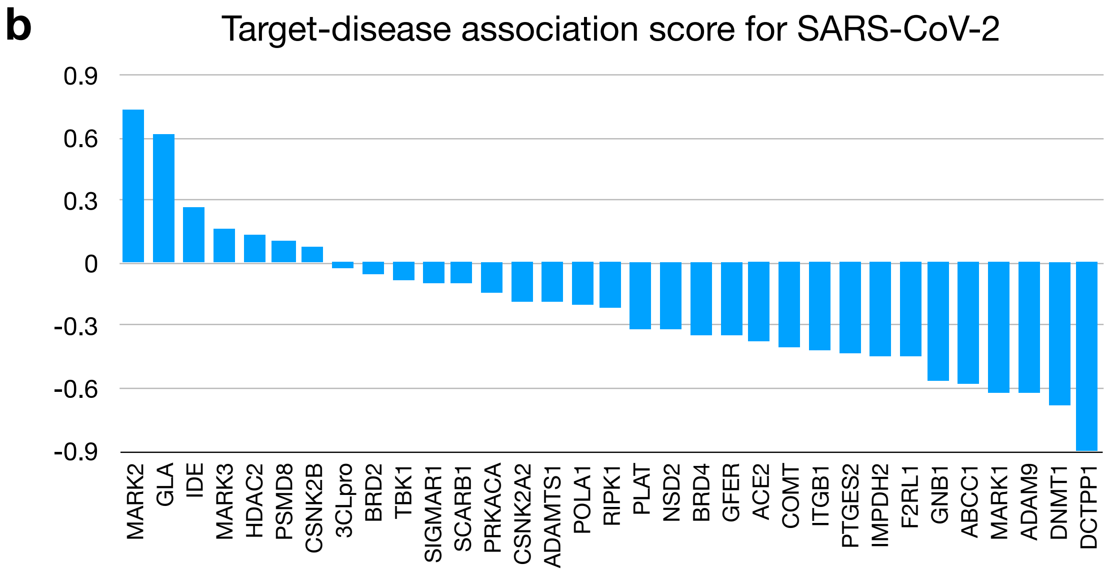

Fig. S1. a) Explanation of predicted synergy between nitazoxanide and remdesivir. The y-axi represents the magnitude of the gradient $\frac{\partial s_{AB}}{z_{A,i}^{covid}}$ (blue bar) and $\frac{\partial s_{AB}}{z_{B,i}^{covid}}$ (green bar) for a biological target $\mathbf{t_i}$. b) Learned target-disease association scores for each biological target related to SARS-CoV-2.

Dataset S1 (separate file). Drug-target interaction data.

Dataset S2 (separate file). SARS-CoV-2 single-agent antiviral activity data.

Dataset S3 (separate file). SARS-CoV-2 drug combination training data.

Dataset S4 (separate file). SARS-CoV-2 drug combination validation data.

Dataset S5 (separate file). SARS-CoV-2 drug combination test data.

Dataset S6 (separate file). HIV single-agent antiviral activity data.

Dataset S7 (separate file). HIV drug combination data.

SI References

1. T. Bobrowski *et al.*, "Synergistic and antagonistic drug combinations against SARS-CoV-2," *Molecular Therapy*. Elsevier, 2020.
2. R. R. Selvaraju, M. Cogswell, A. Das, R. Vedantam, D. Parikh, and D. Batra, "Grad-cam: Visual explanations from deep networks via gradient-based localization," in *Proceedings of the IEEE international conference on computer vision*, 2017, pp. 618–626.
